# Social science research contributions to antimicrobial resistance: protocol for a scoping review

**DOI:** 10.1186/s13643-020-1279-y

**Published:** 2020-02-05

**Authors:** Abou Ali Vedadhir, Carla Rodrigues, Helen Lambert

**Affiliations:** 1 Department of Population Health Sciences, Bristol Medical School, University of Bristol, Canynge Hall, 39 Whatley Road, Bristol, BS8 2PS UK; 2 Department of Anthropology, Faculty of Social Sciences, University of Tehran, Tehran, 14117-13118 Iran

**Keywords:** Social science research, Antimicrobial resistance (AMR), Scoping review

## Abstract

**Background:**

Antimicrobial resistance (AMR) is an escalating global health issue with complex and dynamic interdependencies, high uncertainty and decision stakes, multiple drivers and stakeholders with diverse values and interests, and various aspects and outcomes. Addressing and combating this critical global challenge requires the formation and establishment of an interdisciplinary research approach that goes beyond the biosciences principally concerned with antimicrobial resistance to include other relevant natural and social sciences. The objective of this study will be to review and map existing social science knowledge and literature relating to antimicrobial resistance.

**Methods:**

The review team will undertake the scoping review using the Arksey and O'Malley methodological framework and also the Joanna Briggs Institute methods manual. Publications in English (from 1998 onwards) will be searched using several databases including PubMed/MEDLINE, Web of Science, Scopus, Anthropological Plus, Sociological Abstracts, International Bibliography of the Social Sciences (IBSS), PsycINFO and EconLit. Grey literature will also be searched (e.g. Google Scholar). Two reviewers will independently screen all citations, full-text articles, and abstract data. Publication types will include original articles, editorials, commentaries, protocols, and books in the social science research literature on AMR. All study designs (quantitative, qualitative, and mixed-methods) will be included. A PRISMA Flow Diagram of search and study selection will be used to report final figures on included and excluded studies. To provide a descriptive summary of the literature, data will be collated, stored, and charted using Microsoft Excel software. The analysis will also involve identifying themes and gaps in the existing literature and summarizing, describing and displaying all pertinent information using thematic construction approaches including qualitative content analysis methods.

**Discussion:**

This protocol describes a systematic method to identify, map, and synthesize social science research evidence on antimicrobial resistance. By mapping evidence and identifying potential knowledge gaps where further research is warranted, the resulting scoping review will provide useful insights for the design, implementation, and reorientation of future research agendas on AMR at multiple levels.

Systematic review registration: This protocol has been registered with the Open Science Framework (OSF): https://osf.io/hyaem.

## Background

Antimicrobial resistance (AMR) is widely regarded as one of the escalating global public health challenges of the 21st century [1]. AMR is an evolutionary process whereby microorganisms acquire the ability to withstand antimicrobial drugs, thus making treatment of infections ineffective and increasing the risk of resistant microorganisms spreading among people, animals, and the environment. The global spread of AMR may compromise our ability to treat existing and emerging common infectious diseases, as well as undermine many other improvements in health and sustainable development [2]. “Overuse” and “suboptimal use” of antimicrobials, including antibiotics in humans, farmed animals and the environment, mobility of human populations between regions and healthcare facilities, poor infection control, inadequate sanitary conditions and inappropriate food-handling are considered the main factors leading to the emergence and spread of AMR [3–7].

AMR is a growing, multifaceted public health problem that results in increased prolonged illness, disability and death for patients [4, 8]. Beyond all of these, AMR is widely considered to pose a threat to the future of humanity [9]. According to one estimate, if not curtailed, deaths per year linked with AMR could rise to 10 million by 2050 [10–12]. AMR also increases healthcare costs with lengthier stays in hospitals and a growing requirement for more intensive patient care [4, 12]. It has also been predicted that the mortality from infections subject to AMR could result in a reduction of 2% to 3.5% in global gross domestic product (GDP) in 2050, amounting to between 60 and 100 trillion US dollars worldwide [10, 11, 13]. According to the World Bank [14], people and economies in low- and middle-income countries (LMICs) will experience proportionately greater suffering than high-income countries (HICs) from reduced economic growth and global poverty caused by AMR. Health policymakers recognise that biomedical and life sciences approaches alone are insufficient to address critical global issues as significant and complex as AMR [15]. The adoption of a cross-sectoral ‘one-health’ or ‘eco-health’ approach has therefore been widely advocated [16–19]. Others have argued for a more ‘overtly interdisciplinary’ approach [20] or for an ‘extended peer community’ (engaged citizenry) approach to the issue [21–24], going beyond ‘behaviour’ [25, 26], ‘resistance’ [27], ‘drugs and bugs’ [28], ‘academic tribes and territories’ [29], and/or ‘disciplinary epistemic cultures’ [30, 31].

Together with calls for multidisciplinary and interdisciplinary research, AMR has been characterised as a social rather than a biological problem such that, ‘it demands a social solution, one based on greater understanding, measurement, modelling, and ultimately (re)shaping the social, political, and economic environment in which resistance develops and antibiotics are used’ [32]. There have been numerous calls to address the socio-cultural, economic, and political dimensions of AMR through greater involvement of social scientists in AMR research [25, 26, 33–45].

Yet, despite widespread agreement that social science research could provide vital insights into the sociocultural, economic, political, and organisational determinants and consequences of AMR [41], the social science literature on these issues is sparse and widely scattered [46]. This scoping review, therefore, aims to identify, categorise, summarize, synthesize and map existing knowledge, literature and evidence from published social science research addressing AMR. The available literature will be identified and synthesised in order to provide a map of current knowledge and to identify potential gaps where further social science research is warranted.

## Methods

### Protocol design

The methodological approach draws on a scoping review or scoping study framework. The general purpose of conducting scoping reviews is to identify and map the available evidence and to clarify key concepts/definitions in the literature. It is an ideal tool to determine the coverage of a body of literature on a given topic, or within a research area, and to give a clear indication of the volume of evidence or studies available, as well as a detailed overview of its focus [47–49]. A scoping review can also be used to provide a broad outline of a certain topic or a given field (such as AMR), to examine how research is conducted on a certain topic or field, to develop a “concept map” of a particular concept or approach in the literature—what it refers to, and what it encompasses—and to map evidence in relation to time, location, source (peer-reviewed or grey literature), and/or origin (healthcare or other academic disciplines) [50]. In addition, scoping reviews can be useful to detect and analyse gaps in the existing body of knowledge and literature. They can, moreover, contribute to rapid evidence review in emerging fields or topics or to examine emerging evidence when it is still unclear what other, more specific, questions can be posed and valuably addressed by a more precise systematic review [49].

The present protocol has been registered within the Open Science Framework (registration: https://osf.io/hyaem) and is being reported in accordance with the reporting guidance provided in the Preferred Reporting Items for Systematic Reviews and Meta-Analyses Protocols (PRISMA-P) statement [51, 52] (see checklist in Additional file 1). This study will be conducted as per the methodological framework developed by Arksey and O'Malley [47], the methodology outlined in the Joanna Briggs Institute Reviewers’ Manual [48] and reported as per the PRISMA statement extension for scoping reviews (PRISMA-ScR) [53].

The stages of the study will comprise:

1. Identification of the research questions and objectives
2. Identification of relevant published studies and documents
3. Selection of studies and documents
4. Extracting and charting the evidence and data
5. Collating, summarising and disseminating the results and identifying the implications of the study findings for policy, practice, and research on AMR.

This protocol was drafted using the PRISMA-ScR checklist and explanation, recently developed and revised by an international research team [53] with the aim of providing a rationale and an example of good reporting for each item of a systematic scoping review.

Oversight of protocol development, inclusion/exclusion criteria, and the review process will be provided by a multi-disciplinary advisory group including experts from medical anthropology, sociology, health economics, social psychology, health policy, and health services research.

### Stage one: identification of the research question(s) and objectives

As mentioned above, the main aim of this scoping review is to identify, categorise, summarize, synthesize and map existing knowledge, literature and evidence from published social science research addressing AMR. More specifically, the review will be guided by the following research questions:

- What evidence or studies are available in the social science research literature that address AMR?
- What empirical, conceptual and theoretical elements constitute this body of literature?
- What knowledge and research gaps can be identified in the literature?

### Stage two: identification of relevant published studies/ literature

#### Eligibility criteria

To find and categorise all studies or evidence relevant to this review, the review team will search relevant databases (listed below) of published literature. Outputs will be included if they are: published from 1998 onwards, when the first major UK government review on AMR, “The Path of Least Resistance” was completed [54]; written and published in English only; addressing the phenomenon of evolving microbial resistance to existing antibiotic, antimicrobial or antifungal drugs; using social science approaches and/or methodologies [55], with social sciences defined as the disciplines included in the UK Economic and Social Research Council (UK ESRC)’s list [56]. Publication types will include original research articles, reviews, commentaries, short communications of findings, books and book chapters, protocol papers, theory/discussion papers, and editorials. Book reviews, wiki articles, blogs, websites, practice guidelines, leaflets and brochures, policy statements, reports on scientific meetings, and corporate literature and data will be excluded. All research designs (quantitative, qualitative, and mixed-methods studies) will be included. Studies and documents focused solely on clinical, biomedical, veterinary and microbiological aspects, mechanisms or processes of AMR will be excluded. Studies will also be included if they address human lives or life experiences, practices, organisations or structures associated with AMR and its drivers in non-human settings or subjects (e.g. veterinary or environmental aspects), but studies in animals, plants, or the natural environment will be excluded. As shown in Table 1, the Setting, Perspective, Intervention, Comparison, Evaluation (SPICE) framework [57, 58] is used to develop and outline inclusion/exclusion criteria and to frame the review questions. Reasons for inclusion of all relevant evidence will be documented at full-text review stage.

**Table 1:** Study details, characteristics, and results extraction instrument

Scoping review title:	Social science research contributions to antimicrobial resistance: a scoping review
Review objective/s:	To identify, categorise, summarize, synthesize and map out existing knowledge, literature and evidence on AMR from social sciences research
Review question/s:	• What evidence or studies are available that address social, cultural, organizational, political or economic dimensions of AMR? • What empirical, conceptual and/or theoretical elements constitute this body of literature? • What knowledge and research gaps can be identified?
Concepts (what*):	AMR, Social Sciences
Population (for whom*):	Humans (excluding studies conducted in animals and plants)
Core concept:	Social science research contributions to AMR
Language:	English
Date of publication:	January 1998–September 2019
Data extraction:	Name (i.e., person extracting data)
	Date
Publication details	
Author(s):	
Title:	
Type of publication/source (e.g. commentary/peer-reviewed journal)	
Year and place of publication:	
Aim(s)/research question(s):	
Type of study and/or methodological approach (including data collection methods and analytical approach, if available)	
Academic discipline/disciplinary approach (e.g. sociology, anthropology, economics):	
Location (where*) (e.g. country/province; rural/urban; country income level):	
Context (if applicable) (e.g. patients’ home, primary/secondary/tertiary healthcare, pharmacies/ drug shops, farms, local/national/international policy):	
Sample size (if applicable):	
Year(s) of data collection:	
Other results extracted from study or document content	
Conceptual/theoretical framework or approach:	
Domains addressed/focus of study (e.g., prescribing, consuming or dispensing practices, social interactions including user—prescriber and/or professional—institutional interactions, formal/informal aspects, stockholders, contextual factors, drivers, costs and impacts, socio-cultural meanings, images and stigma, intervention development or evaluation, etc.);	
Key findings that relate to the scoping review question(s) (*what result):	
Comments on gaps, inconsistencies, biases and unmet needs in AMR research:	
Reported AMR-related academic activities (e.g., research and teaching programs, fellowships, funded projects; NGOs and networks; program and policy development, campaigns, advocacy, and knowledge exchange activities, regulation and delivery on AMR, etc.):	
Other emerging information or themes (*what else):	

*Components of the SPICE framework: Setting (where); Perspective/Population (for whom); Intervention/Phenomena of Interest (what); Comparison (what else); Evaluation (what result or how well)

#### Information sources

Potentially relevant studies, published in English between 1st January 1998 and 30th September 2019, will be searched in electronic databases and other relevant grey literature sources. The primary source of literature will be a structured search of major electronic databases and will include: Web of Science Core Collection; PubMed including Medline; Scopus; Sociological Abstracts; The International Bibliography of the Social Sciences (IBSS); Anthropological Plus; PsycINFO and EconLit. The secondary sources for searching grey or difficult to locate literature will include Google (Google Scholar and Google Books), Open Grey, ProQuest, and worldwidescience.org. Hand searches of the reference lists of included studies, reviews or other relevant documents, will be performed to identify additional relevant publications that may not be directly indexed in itemised sources. Content experts and authors who are prolific in the field will also be contacted. The literature searches will be designed and conducted by the review team in collaboration with two trained information specialists. The search will include a comprehensive range of terms and keywords related to social science disciplines, specific approaches and methodologies in social science research, and antibiotic, antimicrobial or drug resistance. For social science disciplines, as mentioned above, the search will adapt definitions from the UK ESRC’s list [56]. A draft search strategy for Web of Science is provided in Additional file 2. The final search results will be exported into EndNote X9 and all duplicates will be detected and then eliminated.

### Stage three: selection of studies and documents

A two-stage selection process will be adopted consisting of an initial screening based on title and abstract only, followed by a full-text review of included items. All titles and abstracts retrieved in the search will be read, reviewed, and validated independently by two members of the research team. Documents not meeting the defined eligibility criteria will be excluded from full-text analysis.

Two reviewers will independently analyse the content of included full-text articles. The selected studies will be reread and re-evaluated by a third reviewer in cases of uncertainty or disagreement, and decisions about the eligibility of a publication resolved through discussion to reach consensus.

### Stage four: extracting and charting the evidence and data

A standardised data abstraction template has been developed, adapted from the JBI Template extraction instrument [48]. The template will be used to extract data from full-text articles and other sources which meet the eligibility criteria (see Table 1). Two members of the research team will undertake data extraction and will review the completed template for each item. Any disagreements will be resolved through discussion and, when necessary, independent validation by the third reviewer (PI).

Piloting of the data abstraction template will be achieved through independent data extraction of three downloaded eligible articles purposively selected to be diverse in content by all members of the research team, followed by comparison and discussion to reach consensus on any modifications needed. At intervals during the data extraction and charting process, charted data will be compared and discussed to ensure consistency between reviewers and to enable iterative reflection on emerging themes and categories.

### Stage five: collating, summarising and disseminating the results and identifying the implications of the study findings for policy, practice and research on AMR

A flow diagram, developed in line with PRISMA-ScR guidance [53], will be used to report the review searching and inclusion/exclusion pathway. To provide a descriptive summary of the literature, data will be collated, stored and charted using Microsoft Excel software. An empirical approach will be taken for descriptive characteristics (e.g. study type, method, location) but a narrative and flexible strategy will be employed in summarizing and synthesizing review findings on domains addressed and conceptual frameworks identified in the retrieved literature. To quantitatively describe characteristics of the included studies, key information or categories will be extracted from each source and presented using a variety of descriptive techniques including summary and frequency tables, graphs (e.g. Bubble Plot) and study matrices. Additionally, the analysis will involve identifying themes and gaps in the existing literature (both quantitative and qualitative) and summarising, describing and displaying all pertinent information using thematic construction approaches including content analysis methods. A narrative approach will be employed to describe themes that emerge from the extracted data; topics will be grouped by meaning and classified into coherent, relevant and clearly defined themes by two reviewers. The results will be compared and consolidated through accord between the two reviewers and the principal investigator (PI).

### Quality assessment and risk of bias

The purpose of this scoping review is to recognise all the existing evidence or studies on the social science research literature that address AMR. As such, study quality or a formal risk of bias will not be assessed, nor will it be used as a basis for exclusion of studies. This is consistent with relevant guidance [47, 53].

## Discussion

By systematically mapping existing social science research evidence on antimicrobial resistance and identifying potential knowledge gaps where further research is warranted, this scoping review will provide useful insights for the design, implementation, and orientation of future multi- and inter-disciplinary research on the global challenge of AMR at multiple levels: local, provincial, national, and global. The methodological process, key results, insights, and implications of this review may be of interest for diverse audiences and stakeholders inside and outside academia, including researchers, academics and policy-makers in a wide range of relevant fields beyond social sciences, given current acknowledgement of the relevance and value of social science research in addressing the complex global problem of AMR.

This review has several potential limitations. Although it acknowledges the need for ‘one-health’ or ‘eco-health’ interdisciplinary approaches to AMR, only publications relating to human populations, behaviours, and experiences (albeit including those which may influence environmental and veterinary aspects of AMR) will be included. Despite following well-recognised sources such as the UK ESRC and drawing on the expertise of researchers from a range of disciplines, determining what constitutes the content and scope of social sciences on the one hand and their contributions to AMR on the other is challenging. Increasing interdisciplinarity in the health sciences and the adoption of methods from the social sciences into a variety of other disciplines add to the difficulties of agreeing terminology and applicability and may result in the inadvertent exclusion of some relevant sources of evidence. Additionally, the lack of clarity in some studies regarding the disciplinary approach being taken may have implications for analytical categorisation of those contributions. The analysis of findings will take all these issues into consideration. The review team also acknowledges that social science research contributions to understanding AMR in societal context are not limited to published papers that directly address the problem of resistance (for instance, the substantial body of sociological and anthropological literature on medicines and pharmaceuticalisation is clearly relevant). A thorough approach to the literature, however, required narrowing down the selection criteria for this scoping review and these related literatures have been widely discussed previously. The review team believes this approach will improve the quality of the outcomes of this project.

Ethical approval is not required as this scoping review solely entails secondary analysis of previously collected and publicly available publications and materials. However, the ethical precepts of copyright and intellectual property will be respected. During the review process, any amendments to the protocol that are deemed necessary by the review team will be recorded in the master protocol document and the reasons for the amendment noted on file; outputs reporting the review results will report any such amendments. The results of this review will be disseminated through peer-reviewed publications and presented at national and international conferences, targeting audiences interested or involved in research into AMR with a social science research approach.

## Supplementary information

**Additional file 1.** PRISMA-P 2015 Checklist.

**Additional file 2.** Search Strategy.

## Data Availability

All data generated or analysed during this study are included in this published article.

## Abbreviations

AMR: Antimicrobial resistance
JBI: the Joanna Briggs Institute
PRISMA-P: Preferred Reporting Items for Systematic Review and Meta-Analysis Protocols
PRISMA-ScR: The Preferred Reporting Items for Systematic reviews and Meta-Analyses extension for Scoping Reviews
