# Supplementary material for: Social science research contributions to antimicrobial resistance: protocol for a scoping review
Source: Syst Rev. 2020 Feb 5;9:24. doi: 10.1186/s13643-020-1279-y (PMC7003437; doi:10.1186/s13643-020-1279-y)
Supplement: Supplementary file 2 — Additional file 2. Search Strategy. [file 13643_2020_1279_MOESM2_ESM.docx]

**Additional File 2 – Search Strategy**

The search strategy consists of subject headings, keywords and related terms for studying AMR from social science research standpoints. Depending on the database, some subject terms will be adjusted, allowing us to reduce the noise in the results and to capture all relevant search topics under a given term, since pilot attempts to apply the same search string to a more interdisciplinary database, such as Web of Science Core Collection, required some procedural adjustments. The search results from each database will be iterated and reviewed by the research team. The following descriptors, keywords and their combinations will be used, guided by Boolean operators AND, OR, NOT or AND NOT, as necessary, to construct a tailored search strategy for the Web of Science Core Collection:

*TS=(("Social Science*" OR Demograph* OR "Social statistics" OR "Development Stud*" OR "medical geograph*" OR "health geograph*" OR "Environmental planning" OR "Health Econom*" OR "Management stud*" OR "Business stud*" OR Education OR Anthropolog* OR Linguistics OR Law OR Histor* OR Politic* OR "International relations" OR Psycholog* OR Sociolog* OR "Science and technology stud*" OR "Communication science*" OR "Cultural Stud*" OR multidisciplinar* OR "cross-disciplinar*" OR interdisciplinar* OR transdisciplinar* OR Ethnograph* OR Ethnomethodolog* OR "Narrative Research" OR Phenomenolog* OR "Grounded Theory" OR Interpretative OR Interpretive OR Hermeneutic* OR "Social Survey" OR "Action Research" OR "Social Construct*" OR Realism OR Realist) NOT ("Patient history" OR "Clinical History") AND ("Antimicrobial Resistan*" OR "Anti-microbial Resistan*" OR "Antibiotic Resistan*" OR "Drug Resistan*"))*
